# A Case of Ovariotomy

**Published:** 1873-12

**Authors:** J. C. Reeve

**Affiliations:** Dayton, O.


### A Case of Ovariotomy.

By J. C. Reeve, M. D., Dayton, O.

Ovariotomy has long since taken its place among legitimate operations and is now too frequently performed to justify publication of itself alone. There are yet, however, many points of detail in regard to which wide differences of opinion exist and which can only be decided by a comparison of very many cases and by the teachings of long experience. It is in this light alone that the following case is presented as a contribution to this subject:

July 25th, 1873, Mary Ryan presented herself at my office for examination, having been sent by her physician, Dr. Spitler, of Salem, in this county, as the subject of ovarian tumor. She will be 17 years of age in August, has always enjoyed good health, is now of good color, and with the exception of the abdominal enlargement, appears to be perfectly well. She began to menstruate at 13, and has been since generally regular. About Christmas, 1871, she first noticed a lump or swelling in the abdomen, in the right iliac region, which was tender to the touch, and she has had more or less pain there for three years past. The tumor has steadily increased in size until the present time.

At present her appetite is good and digestion well performed; bowels generally costive. Pulse 100; quite feeble; in marked contrast in this respect to her general appearance. Some difficulty in respiration when lying down at night. She menstruates every five weeks; quality, duration and amount about as usual with her before this trouble began. The enlargement of the abdomen is somewhat more prominent towards the right side, and it presents an unevenness above the inguinal region; the measurements are, around at the umbilicus 36½ inches, from anterior inferior spinous process of left ilium to umbilicus 8 inches, right, 7¾. There is dullness on percussion all over the front and right side of abdomen; a tympanitic sound is discovered on the left at six inches from the umbilicus. Loins; left, tympanitic; right, dull. Auscultation gives only negative evidence. The tumor is elastic to the touch, but there is no fluctuation to be discovered at any point or in any direction. It is scarcely movable and seems pretty firmly fixed in the abdomen. Vaginal examination; cervix natural, os open, admits tip of finger, uterus movable, apparently not enlarged, the sound passes one inch deeper than normal. No part of the tumor to be felt.

No change in the appearance of the mammary areolæ.

I visited the patient on the 11th of September, and repeated the examination in every particular. She still looked well, but pulse was feeble, 96 standing; 72 lying down. Hands and feet cold. Bowels now regular. The measurements of abdomen are now 39.9 and 8¼ inches. She has had pain for two weeks past in right iliac region. Menstruated three weeks ago. The tumor can now be felt above the pubes per vaginam. No signs of disease of heart, lungs or kidneys.

Diagnosis: multilocular cystic tumor of right ovary.

I may say that her *morale* was excellent. She was calm, cheerful, quite confident of recovery, and after the day was fixed for the operation, she was impatient for its arrival.

Her menstruation began on the 19th of September and closed on the 24th, and she was then placed on careful regimen, and took three times daily ten drops of the tincture muriate of iron, and the same quantity of tinct. nux. vom.*

The operation was performed Sept. 30th. She had had slight diarrhœa following a dose of oil given the day before and for this her physician had given her a grain of opium in the morning. Just before going on the table I administered a hypodermic injection of seven minims of Magendie's solution of morphia containing half a grain of atropia to the ounce. The anæsthetic used was the mixed vapors of alcohol, chloroform and ether, the mixture recommended by the Royal Med. Chir. Society of London, and one in the superior safety of which I have every confidence. It was administered by Dr. E. B. Davis, of Dayton, and I was assisted by Drs. Thos. L. Neal, T. D. Davis, and my partner, W. J. Conklin, and Dr. Spritler. Several other medical gentlemen were present.

* For influence of Nux Vomica in improving capillary circulation see Ringer's Hand-Book of Therapeutics, p. 3[illegible]3.

The incision, at least eight inches in length, extended from the umbilicus to near the pubes and upon opening the abdomen some ounces of ascitic fluid were found. Adhesions existed to the walls of the abdomen in front and on the right side, but they were neither close nor difficult to detach. The first cyst tapped poured out a dark brown liquid, running freely, and showing sparkling crystals of cholesterine on the surface; the contents of the others were too thick to come freely through a canula of three-eighths of an inch diameter, and in one or two it was so tenaceous as scarcely to run at all, but could be pulled out in gelatinous strings. Five cysts were thus emptied in all. Upon getting the tumor out of the abdominal cavity the pedicle was found a long loose leash of vessels already partially stripped from the surface of the tumor. This had undoubtedly occurred from rolling the tumor over in different directions to bring the cysts forward for emptying, and in its final escape from the abdomen, or both combined, and may seem as if imprudent force has been used, yet no strain upon the pedicle was at any time felt. I immediately recognized the fact that I had unconsciously advanced in the process of enucleating the tumor, as first proposed by Dr. Miner, of Buffalo, and the pedicle was then farther stripped and peeled off except at one point where a ligature was placed around a small portion and this divided. I might have thus left it, or perhaps even without this small part ligated, and probably with safety, but I confess my courage failed. In one portion was a vein as large as the handle of a penholder, and upon reflection, I decided to transfix and tie the pedicle in two portions and cut off several inches of the ragged ends. After thorough and careful sponging of the abdominal cavity, and waiting for all bleeding to cease, and after ascertaining that the left ovary was in a healthy condition, the abdomen was closed with silk sutures passed through the peritoneum. The cut end of the pedicle was retained in the abdominal wall by two of the sutures being passed close to it; in other words, the pedicle was "pocketed." A piece of lint wet in warm water was laid over the wound, some cotton batting similarly moistened, above that, then more dry cotton, a piece of rubber cloth above that, and a flannel bandage pinned over all, and she was carried to bed in just an hour from the time of beginning.

She was carefully watched and nursed after the operation, and made a speedy recovery. There were a few attacks of pretty severe colic pain, requiring morphia, and by it soon quieted. The highest febrile movement was on the third day, when the pulse rose to 120 and the temperature to 102.4, and this probably was caused by the sutures which were secured, all but one, on the evening of that day, the time of the first dressing. One of the pedicle ligatures was removed on the 12th of October and the other on the 17th.

Much of the contents of the tumor was lost on account of being so gelatinous in character; the weight of all was estimated, by the medical gentlemen present, to be twenty-five pounds.—*The Clinic*.

